# Supplementary material for: Effects of Harvest Timing on Phytochemical Composition in Lamiaceae Plants under an Environment-Controlled System
Source: Antioxidants (Basel). 2023 Oct 25;12(11):1909. doi: 10.3390/antiox12111909 (PMC10669742; doi:10.3390/antiox12111909)
Supplement: Supplementary file 1 [file antioxidants-12-01909-s001.zip › antioxidants-2661023-supplementary.pdf]

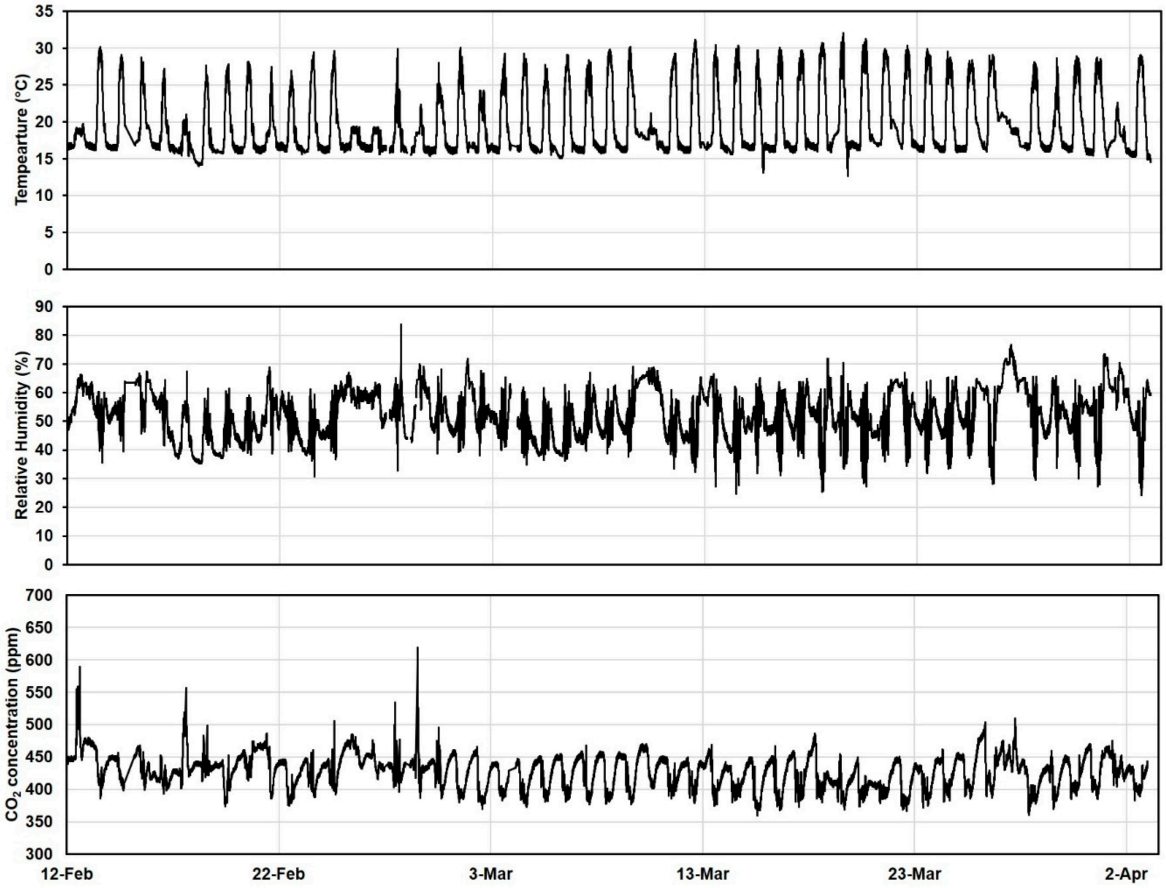

Figure S1. Environmental conditions including (A) temperature (°C), (B) relative humidity (%), and (C) CO_2 concentration (ppm) during the cultivation period.

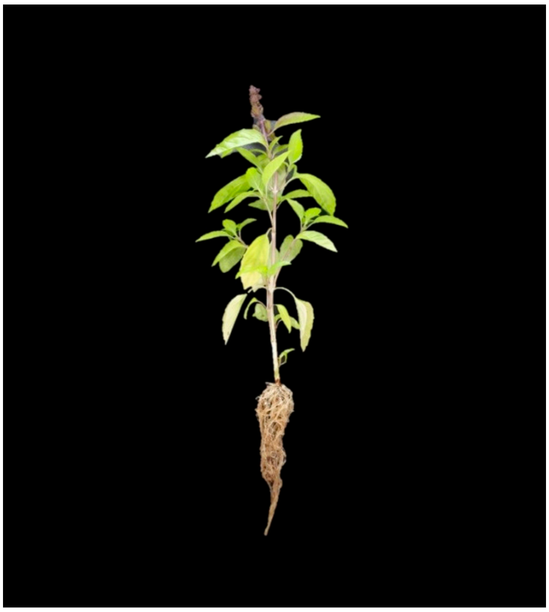

Figure S2: Picture of OBP at 90 DAS.

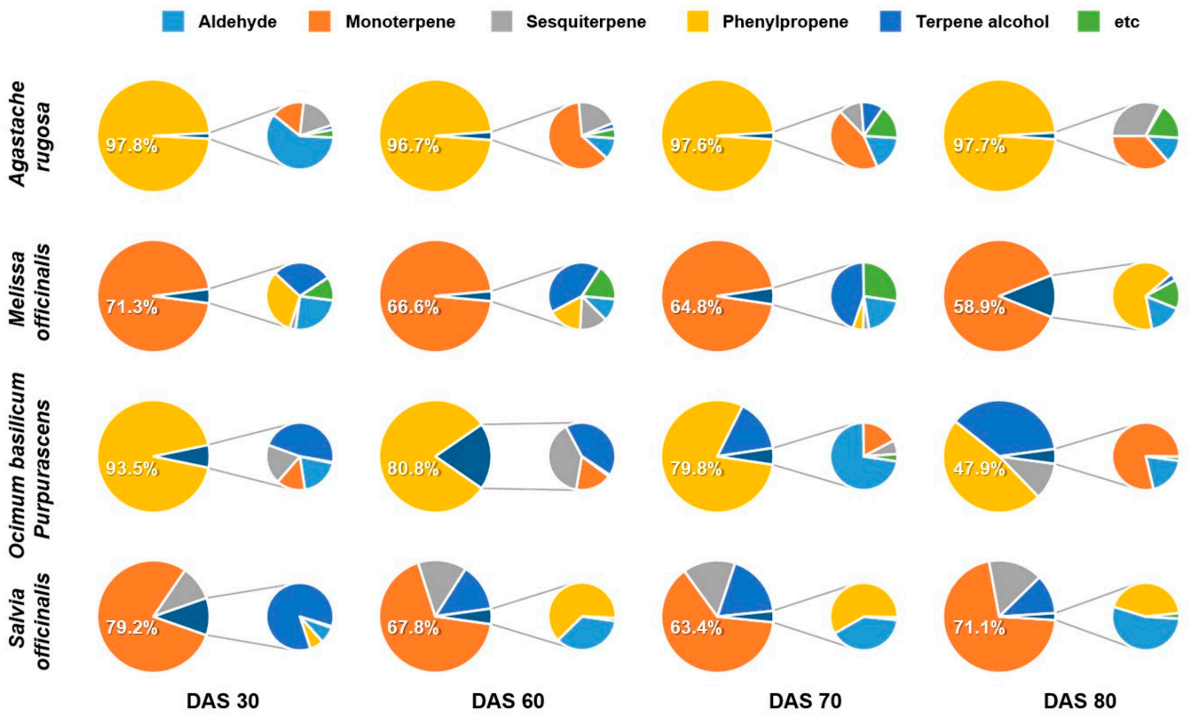

Figure S3. Proportions of volatile organic compound groups (aldehyde, monoterpene, sesquiterpene, phenylpropene, terpene alcohol, and others) metabolites in each plant of (A) AR, (B) MO, (C) OBP, (D) and SO at elevated days after sowing (DAS).
